# Capsaicin-sensitive sensory nerves, TRPV1 receptors and tachykinins play important roles in mast cell tryptase-induced arthritis and hyperalgesia

**DOI:** 10.1186/1471-2210-11-S2-A50

**Published:** 2011-09-05

**Authors:** Zsuzsanna Helyes, Éva Borbély, Katalin Sándor, Adrienn Markovics, Erika Pintér, János Szolcsányi, John P Quinn, Jason J McDougall

**Affiliations:** 1 Department of Pharmacology and Pharmacotherapy, Faculty of Medicine, University of Pécs, 7624 Pécs, Hungary; 2 School of Biomedical Sciences, Liverpool University, Liverpool L69 3BX, UK; 3 Department of Physiology and Pharmacology, University of Calgary, Calgary, Alberta, T2N 4N1, Canada

## Background

Protease-activated receptors (PARs) are G protein-coupled receptors activated through proteolytic cleavage. They are localized on epithelial, endothelial and inflammatory cells, as well as on Transient Receptor Potential Vanilloid 1 (TRPV1) receptor-expressing capsaicin-sensitive sensory nerves. Tachykinins, such as substance P (SP) and neurokinin A (NKA) encoded by the TAC1 gene are released from these fibres and play an important role in inflammatory and nociceptive processes. We investigated the involvement of capsaicin-sensitive peptidergic afferents, TRPV1 ion channels and TAC1-encoded tachykinins in mast cell tryptase (MCT)-induced joint swelling, hyperalgesia and synovial microcirculation.

## Methods

The natural PAR2 activator MCT (20 µl, 12 µg/ml) was injected into the right tibiotarsal joint of mice. Pretreatment with high doses of the TRPV1 receptor agonist resiniferatoxin (RTX) was used to selectively inactivate capsaicin-sensitive peptidergic sensory nerves. TRPV1, TAC1 and neurokinin 1 receptor (NK_1_) gene-deficient animals were also studied compared to their wild-type (WT) C57Bl/6 counterparts. Knee diameter was measured with a digital micrometer, mechanonociceptive threshold with dynamic plantar aesthesiometry and spontaneous weight distribution with incapacitance tester throughout a 6-hour period. Synovial bloodflow in urethane-anaesthetized animals was determined by laser Doppler imaging. In these studies, MCT was applied topically on the joints.

## Results

MCT-induced joint swelling and secondary hyperalgesia were significantly reduced in TAC1^−/−^ and NK_1_^−/−^ mice, but not in the other groups compared to WTs. Spontaneous weight distribution decreased by 10% on the injection site in response to MCT in WT mice, but not in any other groups. Synovial vasodilatation in response to topical MCT application was significantly smaller not only after the destruction of the capsaicin-sensitive afferents by RTX pretreatment, but also by the selective genetic deletion of the TRPV1 ion channels, but was not altered in TAC1 and NK_1_-deficient mice.

## Conclusions

These data provide evidence that MCT-evoked acute oedema and hyperalgesia are mediated by tachykinins through NK_1_ receptor activation. The lack of difference observed in RTX-desensitized and TRPV1^−/−^ mice is likely to be explained by a counteracting effect of simultaneously released inhibitory peptides (e.g. somatostatin, endomorphins) from the same capsaicin-sensitive fibres. In contrast, these afferents and the TRPV1 receptors are essential in acute synovial vasodilatation, but tachykinis are not involved in this response.

